# Fibrinogen; a predictor of injury severity and mortality among patients with traumatic brain injury in Sub-Saharan Africa: a prospective study

**DOI:** 10.21203/rs.3.rs-2596161/v1

**Published:** 2023-03-01

**Authors:** John Baptist Ssenyondwa, Joel Kiryabwire, Martin Kaddumukasa, Devereaux Michael, Larrey Kasereka Kamabu, Moses Galukande, Mark Kaddumukasa, Martha Sajatovic, Timothy Kabanda Makumbi

**Affiliations:** College of Health Sciences Makerere University; Mulago National Referral Hospital; College of Health Sciences Makerere University; Neurological and Behavioral Outcome Center, University Hospitals, Case Medical Center; College of Health Sciences Makerere University; College of Health Sciences Makerere University; College of Health Sciences Makerere University; Neurological and Behavioral Outcome Center, University Hospitals, Case Medical Center; College of Health Sciences Makerere University

**Keywords:** Traumatic Brain Injury, Fibrinogen, Outcomes, Mortality

## Abstract

**Introduction:**

Fibrinogen levels drop quicker than any other factors in severe trauma such as Traumatic Brain Injury (TBI). Contemporaneous studies show that fibrinogen concentrations < 2 g/L are strongly related to mortality. However, little is known regarding fibrinogen levels and TBI severity as well as mortality in sub-Saharan Africa. We therefore set out to determine whether fibrinogen levels are associated with TBI severity and seven days outcomes.

**Objectives:**

To determine the sensitivity and specificity of fibrinogen levels and the association with severity and mortality among TBI patients at Mulago Hospital.

**Methods:**

We prospectively enrolled 213 patients with TBI aged between 13 and 60 years of age and presenting within 24hrs of injury. Patients with pre-existing coagulopathy, concurrent use of anticoagulant or antiplatelet agents, pre-existing hepatic insufficiency, diabetes mellitus and who were pregnant were excluded. Fibrinogen levels were determined using the Clauss fibrinogen assay.

**Results:**

Majority of the patients were male (88.7%) and nearly half were aged 30 or less (48.8%). Fibrinogen levels less than 2g/L were observed in 74 (35.1%) of the patients while levels above 4.5 g/L were observed in 30(14.2%) of the patients. The average time spent in the study was 3.7 ± 2.4 days. The sensitivity and specificity using fibrinogen < 2g/L was 56.5% and 72.9% respectively. Fibrinogen levels predict TBI severity with an AUC = 0.656 (95% CI 0.58–0.73: p = 0.000) Fibrinogen levels < 2g/L (hypofibrinogenemia) were independently associated with severe TBI. (AOR 2.87 CI,1.34–6.14: p = 0.007). Levels above 4.5g/L were also independently associated with injury severity (AOR 2.89, CI 1.12–7.48: p < 0.05) Fibrinogen levels more than 4.5g/L were independently associated with mortality (OR 4.5, CI;1.47–13.61, p < 0.05).

**Conclusions:**

The fibrinogen level is a useful tool in predicting severity including mortality of TBI in our settings. We recommend the routine use of fibrinogen levels in TBI patient evaluations as levels below 2g/L and levels above 4.5g/L are associated with severe injuries and mortality

## Introduction

Trauma accounts for 11% of the world’s disability adjusted life years (DALYs) with 90% of these occurring in Low and Middle Income Countries.(1) Traumatic Brain Injury (TBI) per se is a major cause of disability globally with an incidence rate of 200 per 100 000 people per year(2) In Uganda, head injuries with TBI are the commonest type of injuries accounting for 44% of trauma admissions at hospitals in Kampala(3) and mortality rate of 220/100,000.(4) The morbidity and mortality due to TBI is higher in low-income and middle-income countries(5, 6) despite advancements in the clinical evaluation of patients with TBI using standardized protocols such as the Advanced Trauma Life Support (ATLS) protocols.(7, 8) Evidence from prior studies shows that deaths from trauma can be prevented if adequate and timely identification of the problem is done and the appropriate line of management is decided early.(9) In low and middle income settings where there is limited access to prompt investigation modalities for TBI victims, clinicians often find themselves relying on trauma algorithms, trauma assessment tools and clinical examination findings to diagnose and direct TBI management. Some of the trauma assessment tools employed in the evaluation of TBI patients include; Abbreviated Injury Score(AIS), Trauma Injury Severity Score (TRISS) and the Glasgow Coma Scale (GCS) specifically for TBI.(10) These assessment tools have been found to have considerable limitations and that they may not correlate well with severity of injury.(11) Among these, the GCS remains the commonest tool used to assess TBI severity in Sub-Saharan Africa.(12, 13) Despite its wide utility, the GCS does not provide specific parametric clinical information about the pathophysiologic abnormalities in TBI which are the targets of our interventions.(14) One example of a pathophysiologic event is intracranial bleeding which is also associated with poor clinical outcomes such as mortality and disability.(15)

Fibrinogen, which is a positive acute phase protein(16) as well as a haemostatic protein (17) has been retrospectively studied as a prognostic indicator among TBI patients as well as a predictor of in hospital mortality(18–20). Following trauma, fibrinogen levels deteriorate more frequently and earlier than other routine coagulation parameters.(21, 22) Additionally hypofibrinogenemia has been described as a common occurrence in TBI possibly due to trauma induced coagulopathy.(19, 21, 23) A recent study showed that fibrinogen concentrations less than 2g.L were associated with poor outcomes including mortality in contrast to concentrations above 2.5g/L that are associated with favourable outcomes.(18, 19) Current guidelines also emphasize that fibrinogen concentrations be maintained over 1.5–2.0 g/L in severe trauma patients(22) Fibrinogen, therefore has a pivotal role in TBI; however, little is known regarding its sensitivity and specificity in diagnosis of severe TBI in sub-Saharan Africa.

We therefore set out to study the predictive ability of fibrinogen levels in determining TBI severity and predicting clinical outcomes in TBI as a step in improving prompt diagnosis and management of TBI victims. The aims of the study were to determine the sensitivity and specificity of low fibrinogen levels in predicting severity of traumatic brain injuries, to describe the association of fibrinogen levels with TBI severity and 7-day outcomes among TBI patients at Mulago Hospital. We hypothesized that plasma fibrinogen levels are associated with severity and short-term clinical outcomes in TBI patients.

## Materials And Methods

### Study design and setting.

We prospectively studied 213 randomly selected TBI patients admitted to the Casualty unit at Mulago National Referral Hospital (MNRH) between December 2021 and May 2022. MNRH is the biggest public hospital in Uganda at approximately 5 kilometres from the city centre and it receives 75% of injured victims in Kampala.(24) The Casualty unit of the hospital is the entry point for all trauma cases presenting to the hospital.

### Study population and sampling.

The inclusion criteria were as follows: patients aged 13 to 60 years with a clinical diagnosis of TBI documented using Computed Tomography (CT) or Glasgow Coma Scale (GCS) score by clinician and admitted within 24Hrs of TBI occurrence. The age range of 13 to 60 years was used in consideration of the altered metabolism of fibrinogen that occurs at the young and elderly extremes of age.(25–27) TBI in this study was defined as any alteration of brain function or presence of other evidence of brain pathology based on the GCS or head CT scan in a patient, caused by an external force such as accidents, assault, falls and burns.(28) Patients on concurrent use of anticoagulant or antiplatelet agents, medical diagnosis of liver disease, hypertension, and Diabetes mellitus, patients admitted after 24hrs of the injury occurrence and pregnant women were excluded.

To achieve our first objectives, we used the proportion of patients with low fibrinogen from a prior study (19) and level of precision of 7% at 95% confidence interval to determine a sample size of 186 patients using the Kish and Leslie formula.(29) To study the relationship of fibrinogen with outcomes, we calculated the sample size using formula for cohort studies based on comparison of two proportions representing the event rates in both the exposed and the non-exposed groups.(30) Using proportions from the study by Lv et al.(31), with 95% CI and power of 80%, we determined a sample size of 140 patients for the cohort. Adjusting upwards for losses to follow up, we estimated the sample size to be 200 patients. We therefore enrolled a total of 213 patients using systematic random sampling to answer our objectives. All patients were evaluated and treated according to the local protocol. Informed consent was obtained from the patients included in the study and for the unconscious patients, waiver of informed consent was obtained from the Research Ethics Committee of Makerere University and Mulago Hospital Ethics committee.

### Study procedure and data Collection.

**Figure 1:**
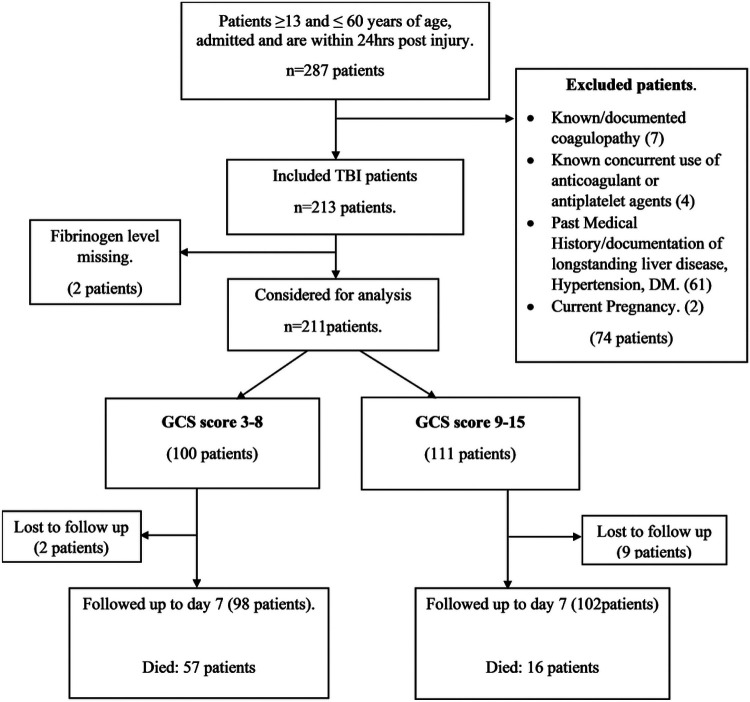
Flow chart showing study procedure and follow up

Data obtained included demographic information such as age, sex, occupation, time of injury, level of education, mechanism of injury and type of head injury. Clinical data including, blood pressure, pulse oximetry, temperature, pupillary reaction, CT scan results, GCS score and fibrinogen levels taken at time of admission were also obtained. The severity of injury was determined by the GCS score obtained by the neurosurgical team. The GCS score of ≤ 8 was categorized as severe TBI and scores 9–15 as non-severe TBI. (Fig. 1) Fibrinogen levels were measured by the Clauss fibrinogen assay using the *“Yumizen G FIB 5”* reagent. The test was carried out on fresh decalcified venous blood obtained from the participants. On admission, 5 milliliters of venous blood were drawn from each of the participants into 3.2% sodium citrate vacutainers and transported to the laboratory within 60 minutes of collection for analysis. Samples were centrifuged to obtain plasma that was prepared for analysis as a 1:10 dilution with Yumizen G IMIDAZOL buffer. The prepared sample was then analysed using an automated analyser and results recorded in g/L. We obtained levels < 1g/L as well as those above 5g/L that were retested at 1:5 dilution and 1:20 dilution respectively to obtain final results. The fibrinogen levels were categorized as: normal fibrinogen levels between 2 and 4.5g/L.(32) A fibrinogen level of < 2 g/L was considered as being low and a level of > 4.5 g/L as high according to standard laboratory reference values. The patients were followed up daily for 7 days and outcomes documented. The clinical outcome studied was in hospital mortality within 7 days of admission. The 11 patients lost to follow up were not analysed for outcomes. (Fig. 1) They were however included in the analysesis for the association between fibrinogen and injury severity on admission to the hospital.

### Statistical analysis.

All study data collected was entered in Epidata version 4.6 software, cleaned and exported to STATA version 14 for analysis. Continuous variables were summarised as means with standard deviation. Categorical variables are expressed as percentages. Bivariate analyses of categorical variables were performed using Pearson’s chi test and presented as p values. A Receiver Operating Characteristic (ROC) curve is used to describe the predictive ability of fibrinogen levels in TBI. The sensitivity ± positive predictive value and specificity ± negative predictive value of fibrinogen levels were calculated using a 2 2 table. Binary logistic regression models were used to describe the relationship between categorical variables between the patient groups with GCS ≤ 8 and that with GCS ≥ 9. Following bivariate analyses, logistic regression multivariate models were used to evaluate the association between fibrinogen and TBI severity as well as in-hospital mortality. All patients with missing data were excluded from the analyses. The models were tested for multiple collinearities, goodness of fit and all independent variables with correlation coefficient above ± 0.4 were excluded from the logistic regression model. The relationship is presented as odds ratio with 95% confidence intervals. All statistical analyses were performed using Stata statistical software, StataCorp. 2015. Stata Statistical Software: Release 14. College Station, TX: StataCorp LP For all analysis, the level of statistical significance was considered when p < 0.05.

## Results

A total of 213 TBI patients were included in the analysis. Road Traffic Crashes (RTCs) were responsible for TBI in 72.3% (n = 154) of the patients followed by assault (n = 45, 21.13%). 101 (47.42%) of the participants had severe TBI while 112(52.58%) had non severe TBI. The majority of the participants were male (n = 189, 88.73%, M: F = 189:24) and aged 30 or less (n = 104, 48.83%). Most of the participants were casual labourers (n = 65, 30.52) and educated to primary school level (n = 99, 46.48%). The average age of the study population was 32.42 ±11.98 years with the minimum age of 13 and maximum age of 60. The majority of patients had closed head injuries (n = 115, 53.99%). The peak time of injury among the patients was during the evening hours between 1700hrs and 2300hrs with 51.5% (n = 103) of injuries during this time of the day. The average time spent in the study by the participants was 3.7 ± 2.4 days. (Table 1) The average length of time spent in hospital before discharge was 4 (± 1.79) days.

The majority 66(31%) of the patients were discharged within 4 days of stay while 21 (10%) of the participants were discharged after 4 days. The average time spent in study before death occurred was 2.1 (± 1.98) days. In the majority (107, 50.71%) of the patients, the fibrinogen levels were between 2–4.5g/L. The maximum level observed was 7.81g/L. The minimum values were as low as < 1g/L. Levels < 2g/L were observed in 74 (35.07%) of the patients. Levels of fibrinogen > 4.5 g/L were observed in 30(14.22%) of the patients. The 7-day mortality rate was 34.3%. Forty-seven patients (47,64.38%) died at Casualty within 24hrs of admission, 14 (19.18%) died in the Neurosurgery unit and 12(16.44%) in the ICU.

## Discussion

This study set out to determine the specificity and sensitivity of fibrinogen levels and the association with severity and mortality among TBI patients at Mulago Hospital. There are no studies addressing this topic from sub-Saharan Africa and this is the first study in Uganda to describe this relationship. Despite numerous evidence available regarding the predictability of fibrinogen in the prognosis of TBI outcomes from prior studies(19, 33), little is known concerning its predictive ability in the diagnosis of severe TBI.

The sensitivity using fibrinogen < 2g/L(hypofibrinogenemia) was 56.5% with a positive predictive value of 64.9%. The specificity was 72.9% with a negative predictive value of 61.7%. In addition, hypofibrinogenemia (fibrinogen levels < 2g/L) was common in TBI patients occurring in 35.07% of TBI patients on admission. This is similar to 38.6% found in a previous study done by Lv, et al.(19) Our study also found that 20.4% of patients with TBI had high levels of fibrinogen(> 4.5g/L).

Recent research showed that for patients admitted with severe TBI, fibrinogen levels < 2g/L on admission are strongly related to increased mortality.(19) In addition, studies have demonstrated that in severe trauma, fibrinogen is reduced to critical levels. (21, 34) The debate about the critical value of fibrinogen in trauma is an ongoing matter of contention.(35) Floccard et’al defined critical levels as being ≤ 1.0g/L and abnormal levels being 1.0–1.8 g/L all of which have been reported in patients with severe Trauma. (36). By contrast, high fibrinogen levels have been described as being protective in patients with multiple trauma.(35) Furthermore, previous studies have demonstrated that TBI is associated with abnormalities in clot formation due to differences in fibrinogen levels among victims.(37) Fibrinogen could therefore be used as a marker or predictor of TBI severity.

The study found that low fibrinogen levels (< 2 g/L) were fairly predictive of TBI severity with an AUC = 0.656, sensitivity of 56.5% and specificity of 72.9%. Therefore, absence of hypofibrinogenemia in TBI patients found in this study was associated with milder forms of TBI. This is consistent with what previous studies have described in severe trauma.

This study shows that the likelihood of having a severe form of TBI increases with low levels of fibrinogen(< 2g/L). Possible explanations for the above relationship stem from the presence of intracranial bleeding which is a common occurrence in TBI as noted in the CRASH trial.(38) Trauma induced coagulopathy is a crucial element in severe TBI especially when compounded with intracranial bleeding. The consumption of clotting factors and platelets in response to intracranial bleeding further lowers fibrinogen levels.(39) TBI is also associated with systemic hyperfibrinolysis which occurs in as much as 20% of critical trauma patients hence lowering fibrinogen levels further. (40, 41) In addition, prior studies have shown that fibrinogen levels drop drastically and most rapidly during haemorrhage.(34) Therefore, the association of low fibrinogen levels with severe TBI is possibly due to a combination of haemorrhage and trauma induced coagulopathy that occurs in severe TBI.(42)

Much as there is a stronger association of severity with low fibrinogen levels, high fibrinogen levels (> 4.5g/L) were also associated with severe TBI. This could be due to the inflammation that accompanies major trauma and disruption of the blood brain barrier with release of procoagulant molecules.(37, 43) A study done by Samuels et al found that TBI patients commonly presented with a spectrum ranging from hypocoagulability to hypercoagulability.(37) It is therefore likely that severe TBI without intracranial haemorrhage leads to high fibrinogen levels while severe TBI with haemorrhage lowers the fibrinogen levels.

Unlike prior research findings, (19) this study showed that fibrinogen levels > 4.5g/L were strong predictors of mortality. A possible explanation of this observation starts with trauma induced disruption of the Blood Brain Barrier that incites an extensive inflammatory response.(16, 44) Such extensive inflammation can be caused by neural cell death with resultant secondary brain oedema.(43) Diffuse Axonal Injury is of utmost importance here since it is characterized by an intense inflammatory response. (45) It is this inflammatory process that is responsible for the elevated fibrinogen levels. Organ dysfunction then ensues from this trauma induced systemic inflammatory state.(46) The development of organ dysfunction is related to the intensity of the trauma induced inflammatory response.(47) Hence, a severe systemic inflammatory response due to a disrupted blood brain barrier causes early organ dysfunction and later multiple organ failure which leads to death.(47, 48). This possibly explains the high fibrinogen levels found to be associated with mortality.

While one of the strengths of our study is that it was carried out prospectively in a high-volume trauma center, some limitations need to be acknowledged. This was a single centre study with a rather small sample size and with limited duration allocated to conduct the study due to specified time frames for research activities. Secondly, fibrinogen is not a routine test in Mulago Hospital for TBI patients and replacement therapy is not currently part of the management protocols in patients with TBI; hence, despite the identification of abnormalities among the participants, correction therapy with concentrate was not possible for the participants. Patients with coagulopathies however received other supplements such as tranexamic acid and Fresh frozen plasma. Additional prospective studies with larger sample size and longer study duration are needed to confirm the predictability of TBI severity and clinical outcomes using fibrinogen levels.

## Conclusions

In conclusion, we established that fibrinogen is a useful tool in predicting severity of TBI and mortality. The study reveals that the sensitivity of fibrinogen levels < 2g/L is 56.5% and the specificity is 72.9%. Fibrinogen fairly predicts TBI severity with an AUC of 0.656.

Fibrinogen levels may be used as an additional tool to screen TBI patients for injury severity. Low fibrinogen levels (< 2g/L) are predictors of TBI severity. High fibrinogen levels > 4.5g/L are also predictors of TBI severity. A fibrinogen level of > 4.5g/L is a strong predictor of mortality in TBI patients. Integrating fibrinogen as a biomarker in TBI management could therefore provide critical information about trauma physiology and ultimately influence clinical decisions. Additional larger prospective studies are needed to confirm these findings.

## Figures and Tables

**Table 1.** Baseline characteristics of the study population.

	GCSSCORE			
	GCS 9–15 n (%)	GCS 3–8	n (%)	p[1]value
N	112(52.58)	101(47.42)		
Age (years)[2]				
13–30	63(58.88)	41 (46.59)		0.0870
31–60	44(41.12)	47(53.41)		
Sex				
Male	100(89.29)	89(88.12)		0.7880
Female	12(10.71)	12(11.88)		
Type of Head Injury				
Closed head injury	58(51.79)	57(56.44)		0.4966
Open head injury	54(48.21)	44(43.56)		
SBP				
90–139mmhg	85(75.89)	58(57.43)		***0.0001***
<90mmhg	9(8.04)	2(1.98)		
>140mmhg	18(16.07)	41(40.59)		
Oximetry[3]				
90–100%	100(91.74)	76(76.77)		***0.0028***
<90%	9(8.26)	23(23.23)		
Temperature(°C)				
36–38	66(58.93)	52(51.49)		***0.0064***
<36	25(22.32)	12(11.88)		
>38	21(18.75)	37(36.63)		
Fibrinogen[4]				
2–4.5g/L	70(63.06)	37(37.00)		***0.0003***
<2g/L	26(23.42)	48(48.00)		
>4.5g/L	15(13.51)	15(15.00)		
Cause of trauma[5]				
RTC	84(75.68)	70(70.71)		0.7713
Assault	21(18.92)	24(24.24)		
Falls	4(3.60)	4(4.04)		
Others	2(1.80)	1(1.01)		
				
Hospital stay (days)	Mean (SD)	Mean (SD)		
Length of stay	3.8 (±2.1)	3.5 (±2.7)		

[1] P value from comparative analysis between severe and non-severe TBI groups. Statistical significance p<0.05

[2] Missing 18

[3] Missing 5

[4] Missing 2

[5] Unspecified cause 3; RTC Road Traffic Crash

**Table 2.** Two by two table showing fibrinogen association with TBI severity.

	GCS score			*Bivariate analysis*		
Fibrinogen^[1]^	GCS≤8	GCS>8	Total	cor ^[2]^	95% CI	*p*^[3]^ value
<2g/L	48(48.00)	26(23.42)	74	3.493	1.876–6.504	***0.000***
2–4.5g/L	37(37.00)	70(63.06)	107	1(Reference)		
>4.5g/L	15(15.00)	15(13.51)	30	1.892	0.834–4.292	0.127
Total	100	111	211			

**Figure 2:**
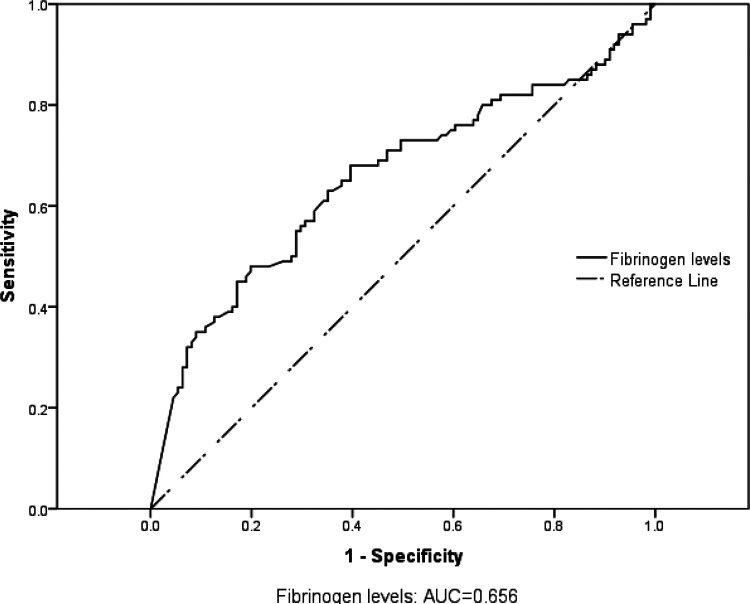
ROC curve showing predictability of fibrinogen levels in TBI severity

The sensitivity using fibrinogen <2g/L was SN =56.5%, PPV=64.9%, (p< 0.001) The specificity using fibrinogen <2g/L was SP =72.9%, NPV=61.7% (p< 0.001). (Table 2) Fibrinogen levels predict TBI severity with an AUC = 0.656, 95% CI (0.58–0.73) p=0.000. (Figure 2)

[1] Missing 2

[2] Crude Odds ratio: Unadjusted for other variables.

[3] Statistical significance determined by chi square test. Values in Bold are statistically significant.

On bivariate analysis, fibrinogen levels were significantly associated with injury severity. SBP and pulse oximetry were also significantly associated with TBI severity. Without adjustment, TBI patients with fibrinogen levels < 2g/L were 3 times more likely to have severe TBI than in participants with fibrinogen levels of 2–4.5g/L. Further multivariate analysis with logistic regression showed a strong association between fibrinogen levels and TBI severity. When adjusted for age, systolic blood pressure, temperature and oximetry; fibrinogen levels < 2g/L (hypofibrinogenemia) were independently associated with severe TBI. (AOR 2.87 CI,1.34–6.14: p = 0.007) Levels above 4.5g/L were also independently associated with injury severity (AOR 2.89, CI 1.12–7.48: p < 0.05) SBP > 140mmHg, oxygen saturation < 90% and temperature > 38° C were also associated with severe TBI. (Table 3)

On bivariate analysis, fibrinogen levels were significantly associated with mortality. Other factors viz SBP temperature and oximetry were also significantly related to mortality. (Table 4)

After adjusting for patient age, hypoxia levels, and hemodynamic status on multivariate analysis, fibrinogen levels more than 4.5g/L were independently associated with mortality (OR 4.5, CI;1.472–13.607, p < 0.05). Temperature > 38°C, SBP and oximetry were also associated with mortality. (Table 4)

**Table 3.** Clinical parameters associated with TBI severity

	GCS SCORE n (%)		Bivariate analysis		Multivariate analysis	
Parameter	GCS 9–15	GCS 3–8	COR[1] (95%CI)	*p*	AOR[2] (95% CI)	*p*
Age (years)^[3]^						
13–30	63(58.88)	41 (46.59)	1(Reference)		1(reference)	
31–60	44(41.12)	47(53.41)	1.64(0.93–2.90)	0.088	1.47(0.75–2.89)	0.261
Sex						
Male	100(89.29)	89(88.12)	1(Reference)			
Female	12(10.71)	12(11.88)	1.12(0.48–2.63)	0.788		
Head injury						
Closed	58(51.79)	57(56.44)	1(Reference)			
Open	54(48.21)	44(43.56)	0.83 (0.48–1.42)	0.497		
SBP						
90–139mmHg	85(75.89)	58(57.43)	1(Reference)		1(reference)	
<90mmHg	9(8.04)	2(1.98)	0.33 (0.07–1.56)	0.161	0.29(0.05–1.66)	0.165
>140mmHg	18(16.07)	41(40.59)	3.34(1.75–6.38)	***0.000***	4.46(1.99–10.00)	***0.000***
Oximetry[4]						
90–100%	100(91.74)	76(76.77)	1(Reference)		1(reference)	
<90%	9(8.26)	23(23.23)	3.36 (1.47–7.68)	***0.004***	2.87(1.02–8.06)	***0.046***
Temperature						
36–38° C	66(58.93)	52(51.49)	1(Reference)		1(reference)	
<36°C	25(22.32)	12(11.88)	0.61 (0.28–1.33)	0.212	0.76(0.31–1.83)	0.534
>38°C	21(18.75)	37(36.63)	2.24 (1.17–4.27)	***0.015***	2.80(1.18–6.64)	***0.02***
Fibrinogen[5] (g/L)						
2–4.5	70(63.06)	37(37.00)	1(Reference)		1(reference)	
<2	26(23.42)	48(48.00)	3.49 (1.88–6.50)	***0.000***	2.87(1.34–6.14)	***0.007***
>4.5	15(13.51)	15(15.00)	1.89 (0.83–4.29)	0.127	2.89(1.12–7.48)	***0.029***

[1] Crude Odds Ratio

[2] Adjusted Odds ratio. Adjusted for age, Systolic Blood Pressure (SBP) Oximetry, temperature. Values in bold italic are statistically significant.

[3] Missing age 18

[4] Missing oximetry 5

[5] Missing fibrinogen 2

**Table 4.** Factors associated with mortality in TBI.

			*Bivariate analysis*		*Multivariate analysis*	
Parameter	Survived n (%)	Died n (%)	COR[1] [95% CI]	*p*	AOR[2]. [95% CI]	*p*
Age(years)^[3]^						
13–30	55(60.44)	28(44.44)	1(reference)		1(reference)	
31–60	36(39.56)	35(55.56)	1.91(1.00–3.66)	0.051	1.78(0.80–3.97)	0.157
Sex						
Male	83(89.25)	63(86.30)	1(reference)			
Female	10(10.75)	10(13.70)	1.32 (0.52–3.36)	0.564		
Head Injury						
Closed Injury	53(56.99)	38(52.05)	1(reference)			
Open injury	40(43.01)	35(47.95)	1.22(0.66–2.26)	0.526		
SBP (mmHg)						
90–139	73(78.49)	42(57.53)	1(reference)		1(reference)	
<90	6(6.45)	1 (1.37)	0.29(0.03–2.49)	0.259	0.24(0.02–2.92)	0.265
>140	14(15.05)	30(41.10)	3.72(1.78–7.80)	***0.000***	4.59 (1.80–11.66)	***0.001***
Temperature °C						
36–38	61(65.59)	34(46.58)	1(reference)		1(reference)	
<36	19(20.43)	9(12.33)	0.85(0.35–2.08)	0.722	1.02 (0.35–2.93)	0.977
>38	13(13.98)	30(41.10)	4.14(1.91–8.98)	***0.000***	4.19 (1.49–11.75)	***0.007***
Oximetry[4]						
90–100%	88(96.70)	51(72.86)	1(reference)		1(reference)	
<90%	3(3.30)	19(27.14)	10.93(3.08–38.74)	***0.000***	9.15 (2.17–38.68)	***0.003***
Fibrinogen[5] (g/L)						
2–4.5	54(58.06)	25(34.72)	1(reference)		1(reference)	
<2	29(31.18)	31(43.06)	2.31(1.15–4.62)	***0.018***	1.64(0.66–4.09)	0.288
>4.5	10(10.75)	16(22.22)	3.46(1.36–8.69)	***0.008***	4.48(1.47–13.61)	***0.008***

[1] Crude Odds Ratio

[2] Adjusted Odds ratio Adjusted for age, Systolic Blood Pressure (SBP) Oximetry, temperature. Values in bold italic are statistically significant

[3] Missing age 18

[4] Missing oximetry 5

[5] Missing Fibrinogen 2

## Data Availability

The datasets generated and/or analyzed during the current study are not publicly available due to confidentiality agreements but are available from the corresponding author on reasonable request.

## Abbreviations

AOR: Adjusted Odds ratio
ATC: Acute Traumatic Coagulopathy
ATLS: Advanced Trauma Life Support
Bpm: beats per minute
COR: Crude Odds ratio
DALYs: Disability Adjusted Life Years
DVT: Deep Vein Thrombosis
EDH: Extradural Hematoma
GCS: Glasgow Coma Scale
ICU: Intensive Care Unit
INR: International Normalized Ratio
IPH: Intraparenchymal Haemorrhage
IVH: Intraventricular Haemorrhage
LAR: Legally Acceptable Representative
LMIC: Low- and Middle-Income Country
LNMP: Last Normal Menstrual Period
MOF: Multiple Organ Failure
RTC: Road Traffic Crash
SBP: Systolic Blood Pressure
SDH: Subdural Hematoma
TBI: Traumatic Brain Injury
TRISS: Trauma and Injury Severity Score
